# Novel Chimeric Protein Vaccines Against *Clostridium difficile* Infection

**DOI:** 10.3389/fimmu.2018.02440

**Published:** 2018-10-22

**Authors:** Shaohui Wang, Yuanguo Wang, Ying Cai, Ciaran P. Kelly, Xingmin Sun

**Affiliations:** ^1^Department of Molecular Medicine, Morsani College of Medicine, University of South Florida, Tampa, FL, United States; ^2^Division of Gastroenterology, Department of Medicine, Beth Israel Deaconess Medical Center, Harvard Medical School, Boston, MA, United States

**Keywords:** *Clostridium difficile* infection, chimeric protein, vaccine, *Salmonella typhimurium* flagellin, immunization

## Abstract

*Clostridium difficile* infection (CDI) is the leading cause of world-wide nosocomial acquired diarrhea in adults. Active vaccination is generally accepted as a logical and cost-effective approach to prevent CDI. In this paper, we have generated two novel chimeric proteins; one designated Tcd169, comprised of the glucosyltransferase domain (GT), the cysteine proteinase domain (CPD), and receptor binding domain (RBD) of TcdB, and the RBD of TcdA; the other designated Tcd169FI, which contains *Salmonella typhimurium* flagellin (sFliC) and Tcd169. Both proteins were expressed in and purified from *Bacillus megaterium*. Point mutations were made in the GT (W102A, D288N) and CPD (C698) of TcdB to ensure that Tcd169 and Tcd169FI were atoxic. Immunization with Tcd169 or Tcd169Fl induced protective immunity against TcdA/TcdB challenge through intraperitoneal injection, also provided mice full protection against infection with a hyper-virulent *C. difficile* strain (BI/NAP1/027). In addition, inclusion of sFlic in the fusion protein (Tcd169Fl) enhanced its protective immunity against toxin challenge, reduced *C. difficile* numbers in feces from Tcd169Fl-immunized mice infected *C. difficile*. Our data show that Tcd169 and Tcd169FI fusion proteins may represent alternative vaccine candidates against CDI.

## Introduction

*Clostridium difficile* (*C. difficile*) is a Gram-positive, spore-forming, toxin-producing and anaerobic bacillus that is transmitted through spore forms (1). It is the most common cause of nosocomial antibiotic-associated diarrhea (2–4). Symptoms of *Clostridium difficile* infection (CDI) range from diarrhea to intestinal inflammation/lesion and death, which are mainly caused by two protein toxins, toxin A (TcdA) and toxin B (TcdB) (5). Globally, CDI remains an urgent public health problem. In the United States, *C. difficile* is the most common healthcare-associated pathogen (6) with approximately half a million infections and more than 29,000 deaths attributable to *C. difficile* per year (7). A recent study showed that mean healthcare costs attributable to primary CDI were $24,205 per patient, and patients with recurrent CDI had an additional $10,580 in infection-related healthcare costs (8). Currently, standard therapy relies on treatment with vancomycin, metronidazole, or fidaxomicin (9–11), but none of which is fully effective, with up to a 35% recurrence rate (12). Treatment of recurrent CDI is one of the major challenges in the field (13–15). Active vaccination is generally accepted as a logical and cost-effective approach to prevent CDI, but more research is needed to determine the clinical benefits of the vaccines (16). Currently, no vaccine is licensed for the prevention of CDI.

Since the major virulence factors of *C. difficile* are TcdA and TcdB (5), tremendous efforts have been made to develop *C. difficile* vaccines targeting both TcdA and TcdB (17–19). However, *C. difficile* survives in environment as spore forms, which are very stable, resistant to antibiotics and harsh conditions, and the root cause of recurrent CDI. Therefore, an ideal and effective *C. difficile* vaccine should target both toxins and *C. difficile* colonization with a goal to prevent toxin-mediated disease symptoms and reduce spore-mediated transmission. In this project, we aimed to construct chimeric proteins containing immunodominant domains/fragments of both toxins and component, which is effective in inducing anti-*C. difficile* colonization immune responses.

Both toxins share similar domain structures (20), including the N terminus catalytic glucosyltransferase domain (GT), the autoproteolytic cysteine proteinase domain (CPD), a central translocation domain (TM), and a C-terminal receptor-binding domain (RBD). Recent studies have indicated that the RBD of TcdB or TcdA can serve as excellent immunogens (20–24). In our previous study (25, 26), and consistent with others (27, 28), we indicated that the N-terminus of TcdB was able to elicit a protective antibody response. We (25) and others (29) also indicated that CPD could play important roles in maintaining the native structure or epitope conformation of GTD. In this study, we generated a new chimeric protein, Tcd169, by fusing GT, CPD, and RBD of TcdB and RBD of TcdA. It has been reported that *Salmonella typhimurium* flagellin (sFliC) protects mice from death during CDI by delaying *C. difficile* growth in the gut (30). SFliC is known potent adjuvant, and is structurally similar to *C. difficile* flagellin FlicC (cFliC) (31). Therefore, we further fused Tcd169 with sFliC, generating Tcd169FI to construct a vaccine candidate targeting both toxins and *C. difficile* colonization/growth. In this communication, we evaluated and characterized the immunogenicity of protective efficacy of these two fusion proteins *in vitro* and *in vivo* (mouse).

## Materials and methods

### Animals

Wild-type C57BL/6 mice were purchased from Charles River Laboratories. Female C57BL/6 mice were housed under the same conditions at a semi-natural light cycle of 14 h:10 h (light: dark) in a specific pathogen-free (SPF) environment. Mice receive water and food *ad libitum*. After infection with *C. difficile*, mice were housed in an infection room. All mouse studies followed the Guide for the Care and Use of Laboratory Animals of the National Institutes of health, and were approved by the Institutes Animal Care and Use Committee (IACUC) at University of South Florida under the protocol number IS00003756. All efforts were made to minimize suffering.

### Preparation of *C. difficile* spores

Sporulation of the *C. difficile* UK1 strain was induced in Clospore medium as described previously (32). Briefly, an overnight 20 ml of *C. difficile* cultured in Columbia Broth was inoculated into 500 ml of Clospore medium, and incubated for 1–2 weeks at 37°C in an anaerobic incubator. The spore suspension was centrifuged at 10,000 g for 20 min, and the pellet was washed five times with sterile water, and suspended in 10 ml of ddH_2_O. The spore suspension was heated at 60°C for 20 min to kill vegetative cells, and stored at 4°C. The spore concentration was determined by serial dilution on TCCFA or BHI plates (33).

### Expression of recombinant fusion proteins Tcd169 and Tcd169FI in *Bacillus megaterium*

We constructed a recombinant fusion protein, containing the GT, CPD, and receptor binding domain (RBD) of TcdB and RBD of TcdA, bridged with a six-amino acid linker (GGSGGS), resulting in protein Tcd169. To generate a vaccine candidate targeting both toxins and *C. difficile* colonization/growth, we further fused Tcd169 with sFliC bridged with the six-amino acid linker (GGSGGS), resulting in protein Tcd169Fl. The chimeric DNA encoding Tcd169 or Tcd169FI was ligated into *Bacillus megaterium* expression vector pHis1525, which adds a C-terminal His-tag to the chimeric proteins. *B. megaterium* is a gram-positive environmental microbe. The protein expressed from *B. megaterium* system can be free of LPS. Tcd169 and Tcd169FI were purified from bacterial lysate by Ni-affinity chromatography followed by size exclusion chromatography (gel filtration) using Superdex 200 column (cat# 28-9909-44, GE Health).

### Western blot analysis

Purified Tcd169 and Tcd169FI proteins were subjected to 8% SDS-PAGE separation. Then, proteins were transferred onto the Nylon membrane. After blocking for 1 h at room temperature with 5% skim milk, the membrane was incubated overnight at 4°C with anti-TcdA, anti-TcdB, or anti-sFliC antibody (Cat: 629701, Biolegend, Bath, UK). After washing with PBST (PBS with 0.05% Tween), the membrane was incubated with horseradish peroxidase-conjugated secondary goat anti-mouse antibody (Cat: ab97023, Abcam, Cambridge, MA), the antibody-reactive bands were revealed by enhanced chemiluminescence detection on Hyperfilm (Thermo Fisher Scientific, Waltham, MA).

### Mouse immunization and subsequent infection with *C. difficile* spores or challenge with TcdA/TcdB

Female C57BL/6 mice were housed under the same conditions. Mice (*n* = 20) were immunized three times at 14-days intervals via i.m. route with 10 μg of Tcd169FI or Tcd169 in phosphate-buffered saline (PBS) along with alum as an adjuvant for each injection (34). Control mice (*n* = 20) only received PBS with alum. Sera were collected.

Fourteen days after the third immunization, immunized and control mice (*n* = 10) were given a mixture of five antibiotics including kanamycin (0.4 mg/ml), gentamycin (0.035 mg/ml), colistin (850 U/ml), metronidazole (0.215 mg/ml), and vancomycin (0.045 mg/ml) in the drinking water for 4 days. After 4 days of antibiotic treatment, all mice were given autoclaved water for 2 days, followed by a single dose of clindamycin (10 mg/kg) intraperitoneally (i.p.) 1 day before challenge with 10^6^ *C. difficile* UK1 (35) spores/mouse by gavage. During the antibiotic pretreatment, food, water, bedding, and cages were autoclaved. Animals were monitored daily for weight changes, diarrhea and survival, and moribund animals were euthanized. The fecal samples were collected on days 0, 1, 3, and 5 post-challenge. Diarrhea was defined as wet tails, loosen or watery feces. The death included the numbers of mice died after infection and mice euthanized if weight loss was >20%.

The remaining 10 mice from Tcd169-/Tcd169Fl-immunized group or control group were i.p., challenged with lethal dosages of TcdA (200 ng/moue, *n* = 5 for each protein group) or TcdB (100 ng/mouse, *n* = 5 for each protein group), monitored for survival and disease symptoms for 80 h.

### ELISA for anti-toxin/sFliC IgG/IgA titers

ELISAs were performed as previously described (25). Briefly, costar 96-well ELISA plates were coated with 100 μl/well of TcdA (0.5 μg/ml), TcdB (0.5 μg/ml), or sFliC (0.5 μg/ml) at 4°C overnight. Following washing of the unbound material, plates were blocked with 300 μl of blocking buffer (PBS + 5% dry milk) at RT for 2 h. After washing, 100 μl of 10-fold serially diluted sera or fecal samples were added into each well of the plates, and incubated for 1.5 h at RT. Following washing with PBS, 100 μl of mouse IgG-HRP (1:3,000) or mouse IgA-HRP (1:3,000) were added to each well, and incubated for 30 min to 1 h. Subsequent to a washing step with PBS, substrate TMB was added to allow color development at room temperature for 5–30 min. The reaction was stopped by addition of H_2_SO_4_ to each well, and the OD values at 450 nm were recorded by a spectrophotometer. Anti-toxin/-FliC IgG or IgA titer of a given sample (serum or fecal samples from immunized mice) was defined as the dilution factor at which the OD_450nm_ is greater than or equal to that of serum or fecal sample from non-immunized mice.

### Neutralizing assay

Mouse intestinal epithelial CT26 cells were used to assess *in vitro* neutralizing activities of serum samples. The neutralizing titer is defined as the maximum dilution of the samples that blocks cell rounding induced by toxin at a given concentration. This given concentration is four times the minimum dose of the toxin that causes all cells round after a 24 h exposure to the toxin, i.e., 1.6 and 0.04 ng/ml for TcdA and TcdB, respectively.

### Measurement of antitoxin IgG isotypes

IgG1, IgG2a, IgG2b, IgG2c, and IgG3 anti-TcdA/B concentrations in the sera of Tcd169- or Tcd169Fl-immunized mice were determined by ELISA using biotinylated anti-mouse IgG isotype antibodies.

### Determination of anti-glucosyltransferase activity of TcdB imposed by sera from Tcd169- or Tcd169Fl-immunized mice

Glucosyltransferase (GT) activity of TcdB was measured by its ability to glucosylate Rho GTPase Rac1 in cell lysates (36). CT26 cell pellets were resuspended in a reaction buffer (50 mM HEPES, pH 7.5, 100 mM KCl, 1 mM MnCl_2_, and 2 mM MgCl_2_), and lysed by passing through a 30 G needle for 40 times. After centrifugation (16,700 g, 3 min), the supernatant was used as a cytosolic fraction (protein concentration 2.5 mg/ml). To perform the glucosylation assay, the cytosolic fraction was incubated with TcdB at 10 ng/ml (with or without serum, sera were diluted at 1:200) at 37°C for 60 min. The reaction was terminated by adding SDS-sample buffer, and samples were heated at 100°C for 5 min before loading on a 12% SDS-PAGE gel. An antibody that specifically recognizes the non-glucosylated form of Rac1 (clone 102, BD Bioscience), anti-β-actin (clone AC-40, Sigma), and HRP-conjugated anti-mouse-IgG (Amersham Biosciences) were used for Western blotting.

### *In vitro* TcdB autoproteolysis assay

The autoproteolysis assays were performed in 25 μl of 20 mM Tris-HCl pH 8.0, containing 0.2 μg of TcdB (37), and the indicated concentration of Inositol hexakisphosphate (InsP6) to induce cleavage. Unless otherwise indicated, the samples were incubated at 37°C for 1 h, then boiled for 5 min in SDS sample buffer containing β-mercaptoethanol (BME) to halt the reaction. Samples were then separated by 8% SDS-PAGE, and the toxin fragments visualized by Coomassie blue staining. Preserum and serum of Tcd169- or Tcd169FI-immunized mice were diluted at 1:200 in the autoprocessing reactions.

### Binding of toxins to CT26 cells

CT26 cells were exposed to the TcdA or TcdB at 10 μg/ml with or without preserum or serum from Tcd169- or Tcd169Fl-immunized mice at 4°C, for 30 min, after being washed three times, cells were collected for Western Blot analysis using anti-TcdA or anti-TcdB antibodies. Preserum and serum of Tcd169- or mTcd169FI-immunized mice were diluted at 1:200 in the binding reaction system.

### Quantification of *C. difficile* spores in mouse feces

Fecal samples were collected on days 0, 1, 3, and 5 post-infection. Fifty milligrams of feces were dissolved with 500 μl sterile water for 16 h at 4°C, and then treated with 500 μl of purified ethanol (Sigma-Aldrich) for 60 min at room temperature to kill vegetative cells. Samples were vortexed, serially diluted, and plated onto selective medium (TCCA) supplemented with taurocholate (0.1% w/v), cefoxitin (16 μg/mL), and L-cycloserine (250 μg/mL). The plates were incubated anaerobically at 37°C for 48 h, colonies counted, and results expressed as the CFU/gram of feces.

### Quantitation of *C. difficile* toxins in mouse feces

After challenges with *C. difficile* spores, feces were collected, and dissolved in sterile PBS (0.1 g/ml) containing protease inhibitor cocktail, and the supernatants were collected after centrifugation and stored at −80°C. TcdA/TcdB concentrations in fecal samples were determined by ELISA. Briefly, 96-well microplates were coated with 100 μl of anti-TcdA (1 μg/ml) or anti-TcdB antibody (1 μg/ml) overnight in PBS at 4°C. On the next day, each well was blocked with 300 μl of blocking buffer (PBS + 5% dry milk) at RT for 2 h. Next, standards and samples were added to each well (100 μl) in duplicate, and incubated for 90 min at 25°C. After another set of washes, HRP-chicken anti-TcdA or anti-TcdB (1:5,000 dilution in PBS, Gallus Immunotech, Shirley, MA) was added to wells for 30 min at RT. A final set of three washes preceded the addition of the TMB Microwell Peroxidase Substrate for 20 min at RT in the dark. The reaction was stopped with 2 N H_2_SO_4_, and the absorbance was measured using a plate reader at 450 nm.

### mTLR5 activation assay

The ability of FliC and Tcd169FI to activate TLR5 was determined using a reporter assay system as previously described (38, 39). In brief, HEK-Blue mTLR5 cells (Invivogen, San Diego, CA) were plated in HEK-Detection Medium at a concentration of ~25,000 cells per well (96-well plate) in the presence of sFliC, Tcd169FI, Tcd169, or H_2_O. After incubation overnight at 37°C, absorbance at 620 nm was measured correlating to TLR5 activation.

### Statistical analysis

Data were analyzed by Kaplan-Meier survival analysis with a log rank test of significance, by analysis of variance (ANOVA), and by one-way or two-way ANOVA followed with Bonferroni posttests using the Prism statistical software program. Results are expressed as means ± standard errors of means. Differences were considered statistically significant if *p* < 0.05.

## Results

### Construction and purification of Tcd169 and Tcd169Fl

**Figure 1:**
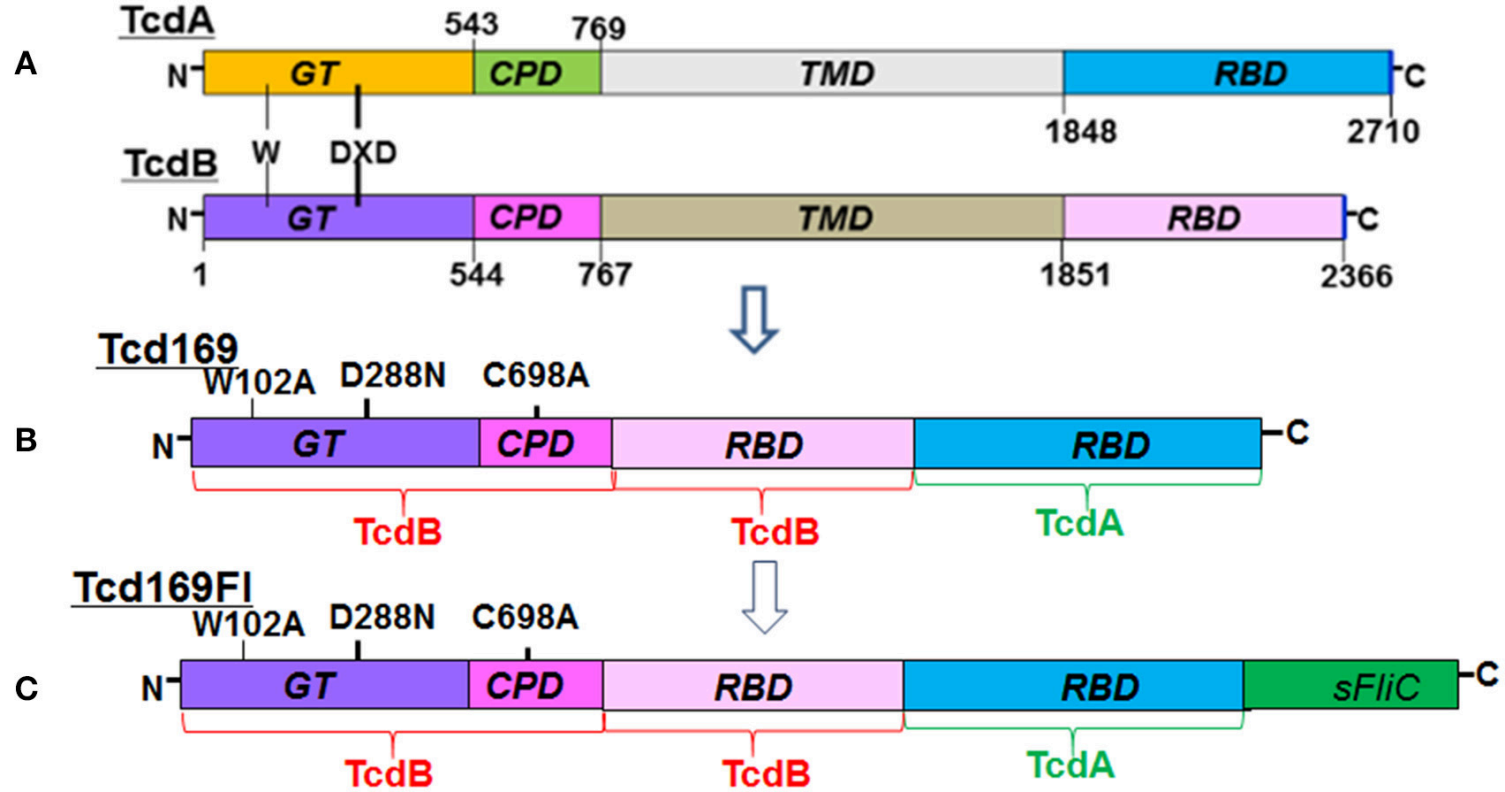
Domains of TcdA and TcdB and construction of Tcd169 and Tcd169FI. **(A)** Both toxins share similar domains, including the glucosyltransferase domain (GT), the cysteine proteinase domain (CPD), the translocation domain (TMD), and the receptor binding domain (RBD). The DXD (D286-x-D288) motif and a conserved tryptophan in the GT are involved in the enzymatic activity. **(B)** Tcd169 was constructed by fusing the GT, CPD, and RBD of TcdB with the RBD of TcdA. Two point mutations were made in the GT of TcdB and one point mutation was made in the CPD of TcdB; these mutations essentially eliminate the toxicity of Tcd169 and Tcd169FI. **(C)** Tcd169FI was constructed by fusing the *Salmonella typhimurium* flagellin (sFliC) with Tcd169.

**Figure 2:**
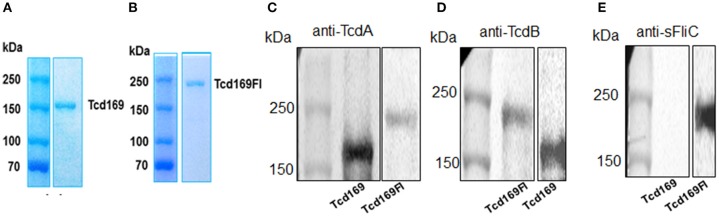
Expression and purification of Tcd169 and Tcd169Fl. Gene sequences encoding Tcd169 and Tcd169Fl were synthesized and cloned in *Bacillus megatarium*. Proteins Tcd169 **(A)** and Tcd169Fl **(B)** were purified from bacterial lysates by Ni-affinity chromatography and gel filtration, and analyzed by SDS-PAGE. Western blot analysis of Tcd169 and Tcd169Fl using anti-TcdA antibody **(C)**, anti-TcdB antibody **(D)**, or anti-sFliC antibody **(E)**.

The RBDs of TcdB and TcdA are highly immunogenic (20, 22, 23). In our previous study (25, 26), and consistent with others (27, 28), we showed that GTD of TcdB was able to elicit a protective antibody response. We (25) and others (29) also found that CPD could play important roles in maintaining the native structure or epitope conformation of GTD. To enhance the immunogenicity of the immunogen, we fused GT, CPD, and RBD of TcdB and RBD of TcdA, resulting in Tcd169 (Figure 1). sFliC is a known potent adjuvant and protects mice from death during CDI by delaying *C. difficile* growth in the gut (30). Therefore, we fused Tcd169 with sFliC, resulting in protein Tcd169FI. The DXD (D286-x-D288) motif and a conserved tryptophan in the GT are involved in the enzymatic activity (40). The cysteine at the position 698 is a critical amino acid mediating CPD activity (40, 41). To ensure that Tcd169 and Tcd169FI were atoxic, point mutations were made in the GT (W102A, D288N) and CPD (C698A) of Tcd169 and mTcd169FI (Figure 1). Recombinant Tcd169 or Tcd169Fl with a 6xHis-tag was expressed in *Bacillus megaterium*, and purified by Ni-affinity chromatography followed by ion exchange purification. The purification process yielded a highly pure product of about 169 kDa (Tcd169, Figure 2A) or 211 kDa (Tcd169FI, Figure 2B). Western blot analysis using specific antibodies against TcdA, TcdB, or sFliC verified the presence of TcdA (Figure 2C), TcdB (Figure 2D), or sFliC (Figure 2E) fragments in Tcd169 or mTcd169Fl.

### Immunizations of mice with Tcd169 or Tcd169Fl induce potent IgG antibody responses against TcdA/TcdB/sFlic, and protect mice against systemic toxin challenge

**Figure 3:**
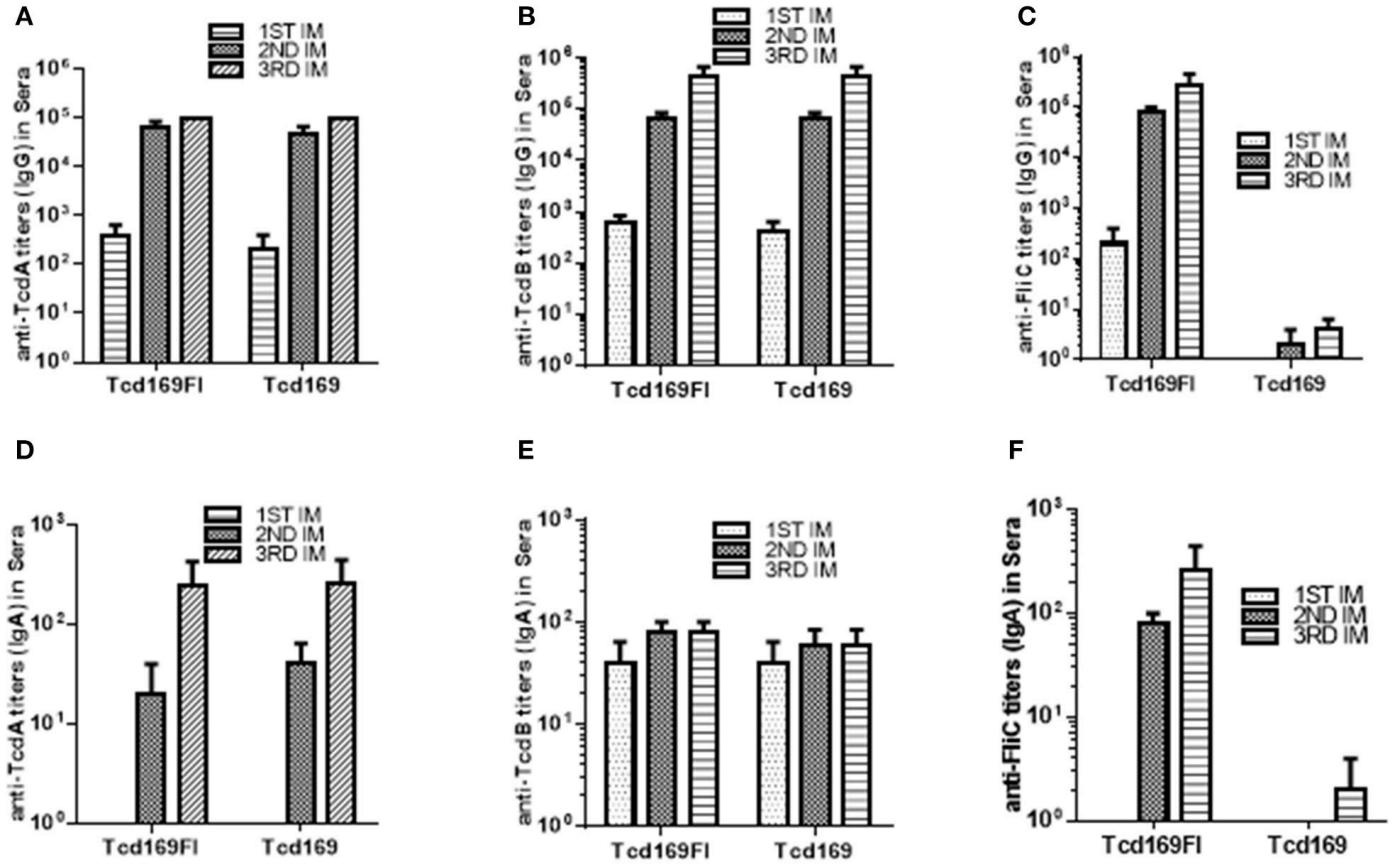
Tcd169 and Tcd169Fl immunizations via intramuscular (i.m.) route induce similar levels of anti-TcdA/anti-TcdB antibodies in sera. Groups of C57BL/6 mice (*n* = 10) were immunized three times at 14-days intervals via i.m. route with 10 μg of Tcd169 or Tcd169FI in combination of alum as an adjuvant. Sera were collected, and anti-TcdA **(A)**, anti-TcdB **(B)**, or anti-sFliC **(C)** IgG titers measured by standard ELISA. Anti-TcdA **(D)**, anti-TcdB **(E)**, or anti-sFliC **(F)** IgA titers were also measured by standard ELISA. Results are given as mean ± SD.

**Figure 4:**
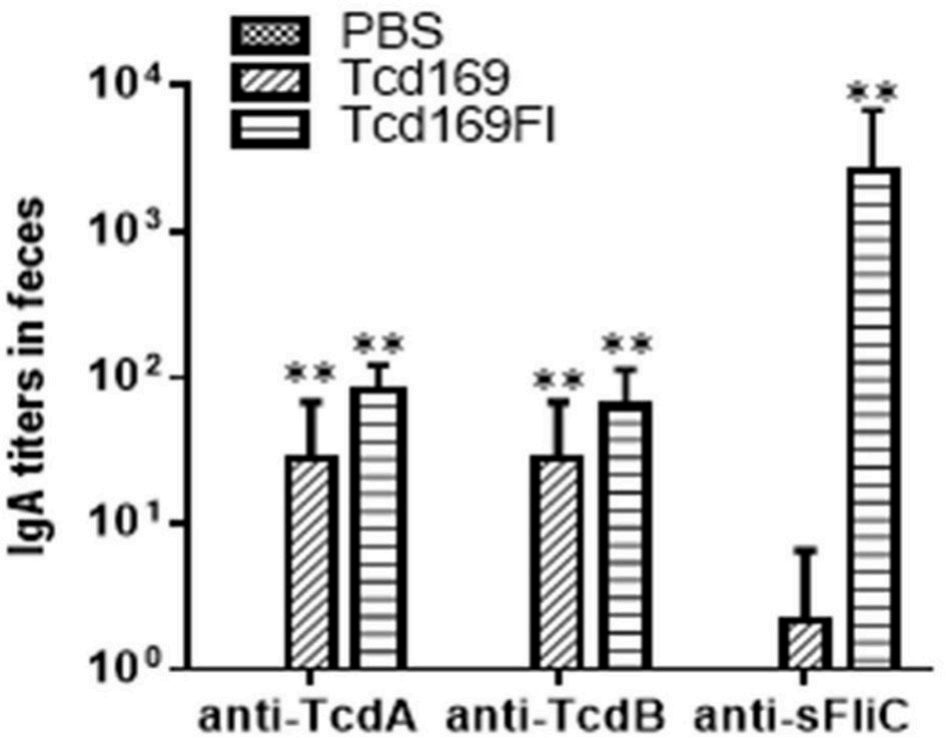
Anti-TcdA/anti-TcdB/anti-sFlic IgA antibodies in feces of mice immunized with Tcd169/Tcd169FI. Groups of C57BL/6 mice (*n* = 10) were immunized three times at 14-days intervals via i.m. route with 10 μg of Tcd169 or Tcd169FI in the presence of alum as an adjuvant. Feces of mice from 3rd immunization were collected, and anti-TcdA, anti-TcdB, or anti-sFliC IgA titers in feces were measured by standard ELISA. Results are given as mean ± SD (**p < 0.01 vs. PBS; *p* < 0.05, between anti-sFliC and anti-TcdA/anti-TcdB titers).

Immunization of mice with 10 μg Tcd169 or Tcd169Fl in combination with alum as an adjuvant via i.m. route induced similar levels of IgG and IgA antibody responses in sera to both toxins (Figures 3A,B,D,E). In addition, Tcd169Fl was also able to induce anti-sFliC IgG/IgA antibody responses in sera (Figures 3C,F). Significant and strong anti-TcdA, anti-TcdB or anti-sFlic IgG responses were induced in the first or second immunizations. Anti-TcdA/-TcdB/-sFlic IgA antibodies in feces of mice immunized with Tcd169/Tcd169FI were also detected (Figure 4). Interestingly, Tcd169Fl was able to induce much stronger anti-sFlic IgA responses than anti-TcdA/anti-TcdB IgA responses (*p* < 0.05).

**Figure 5:**
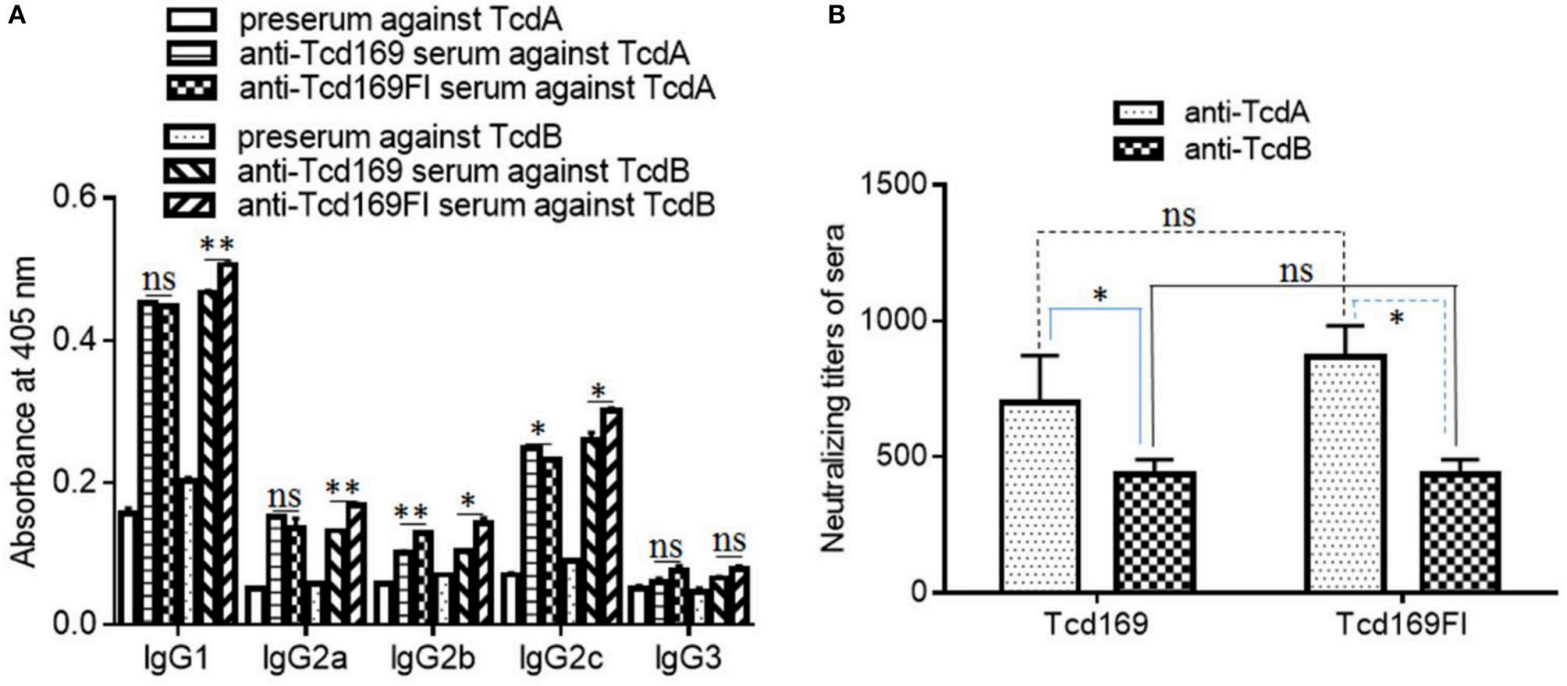
Anti-toxin IgG isotypes and anti-toxin neutralizing titers of sera from mice immunized with Tcd169 or Tcd169FI. Mice were immunized with Tcd169 or Tcd169FI at 10 μg/mouse for three times, and serum samples were collected. **(A)** The anti-toxin IgG isotypes of the serum samples were measured using standard ELISAs. **(B)** Anti-toxin neutralizing titers of sera from Tcd169-immunized or Tcd169FI-immunized mice. Results are given as mean ± SD (*n* = 5) (^*^p < 0.05, ^**^p < 0.01, ns: no significant).

In mice, IgG1 antibody is associated with Th2-like response, and IgG2a, IgG2b, IgG2c, and IgG3 antibodies are associated with Th1-like response (42, 43). Each IgG subclass can participate in the remove of the encapsulated pathogen by distinguished mechanisms. IgG2a and IgG2b show strongest binding to Fc receptors (44) and together with IgG3 fix complement better than IgG1 does; both IgG3 and IgG1 can cooperatively bind to bacteria. Therefore, an immune response with a broad subclass distribution would be useful against encapsulated pathogen. To determine the nature of immune responses (i.e., Th1 or Th2) elicited by Tcd169 or Tcd169Fl immunization, we measured isotypes of anti-TcdA/anti-TcdB IgGs. As shown in Figure 5A, at a dilution of 1 × 10^3^, both anti-Tcd169 and anti-Tcd169Fl sera showed high levels of IgG1 and IgG2c subclass antibodies and significant amounts of IgG2c, IgG2a, and IgG2b, indicating that Tcd169 and Tcd169Fl immunizations can induce both Th1 and Th2 responses with the latter one being stronger. It was reported that *Salmonella typhimurium* flagerlin C (sFliC) has a potent adjuvant property and induces a Th2 response (45). Interestingly, we found that inclusion of sFliC in the Tcd169Fl induced significantly more anti-TcdB IgG subclass antibodies (IgG1, IgG2a, IgG2b, IgG2c) than Tcd169 did (Figure 5A); however, Tcd169Fl did not induce more anti-TcdA IgG subclass antibodies except IgG2b when compared with Tcd169 (Figure 5A).

The ultimate goal of vaccination targeting TcdA/TcdB is to illicit not only high-level anti-TcdA/TcdB antibodies but also potent toxin-neutralizers. Previously, we reported that not all anti-toxin antibodies are toxin-neutralizers, instead some of them are toxin-enhancers (46). Therefore, we also determined the *in vitro* toxin-neutralizing activities of anti-Tcd169 and anti-Tcd169Fl sera. As shown in Figure 5B, both Tcd169 and Tcd169Fl immunizations induced potent neutralizing antibodies against both TcdA and TcdB, with anti-TcdA neutralizing antibody titers being significantly higher than anti-TcdB neutralizing antibody titer in both anti-Tcd169 and anti-Tcd169Fl sera. However, it seemed that Tcd169 and Tcd169Fl induced comparable levels of anti-TcdA and anti-TcdB neutralizing antibodies.

**Figure 6:**
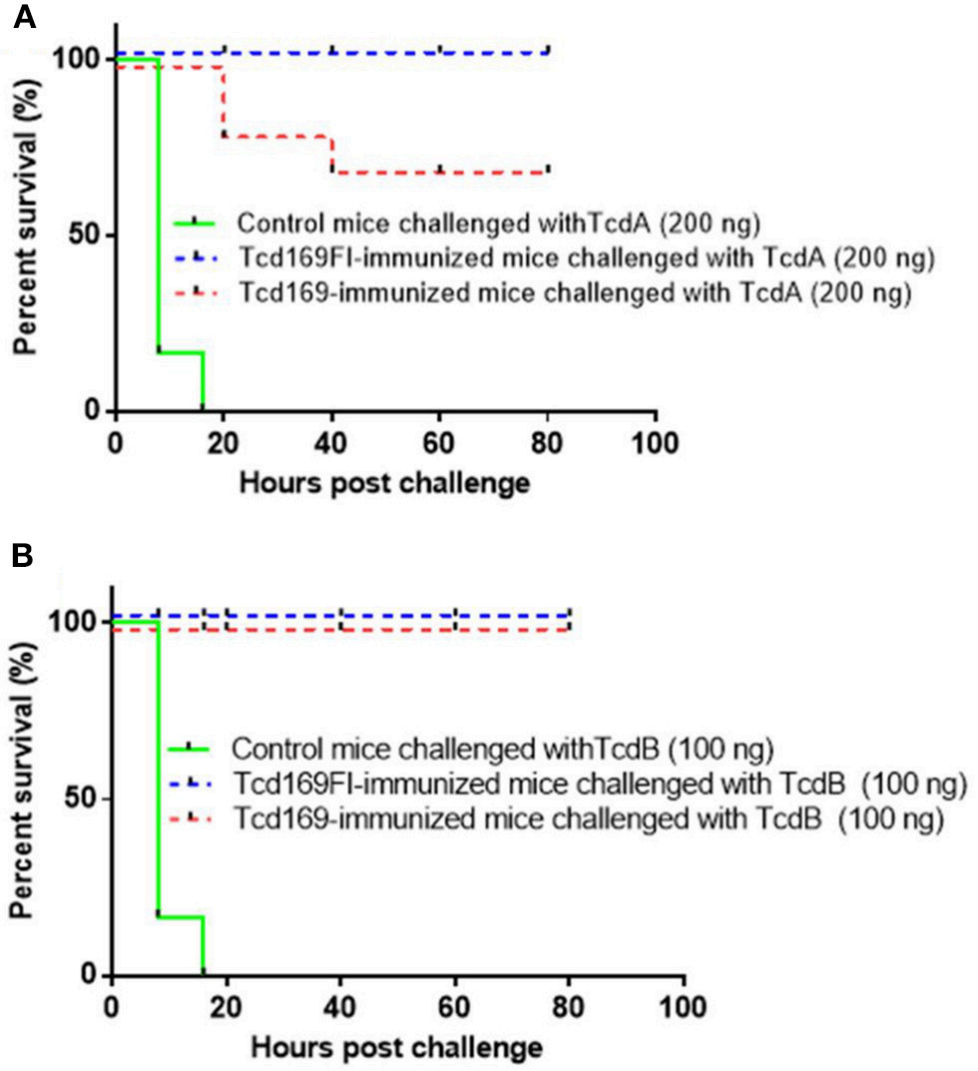
Tcd169/Tcd169Fl immunization protects mice against systemic toxin challenge. Fourteen days after third immunization, Tcd169- or Tcd169Fl- immunized group or control group (*n* = 5) were i.p., challenged with lethal dosage of TcdA (200 ng/mouse) **(A)** or TcdB (100 ng/mouse) **(B)**, and monitored for survival and disease symptoms for 80 h. Kaplan-Meier survival plots of different groups of mice were shown [*P* = 0.0779 between Tcd169- and Tcd169Fl-immunized groups in **(A)**].

To assess the *in vivo* antitoxin neutralizing activities induced by Tcd169 or Tcd169 immunizations. After three immunizations with 10 μg of Tcd169 or Tcd169Fl with alum as an adjuvant, immunized mouse groups (*n* = 5) and control no-immunized mouse groups (*n* = 5) were challenged with lethal doses of TcdA (200 ng/mouse) or TcdB (100 ng/mouse), and mice were monitored for 80 h for survival and other disease symptoms. Immunization of mice with Tcd169 or Tcd169Fl provided full protection against systemic challenge of lethal dose of TcdB (100 ng) (Figure 6). Tcd169Fl immunization also provided mice full protection against TcdA (200 ng) challenge, while Tcd169 immunization only provided partial but significant protection against TcdA challenge, indicating sFliC portion of the Tcd169Fl may enhance the neutralizing activity of anti-TcdA antibodies *in vivo*.

### Glucosyltransferase and cysteine proteinase activities of TcdB are inhibited by anti-Tcd169 or anti-Tcd169Fl serum

**Figure 7:**
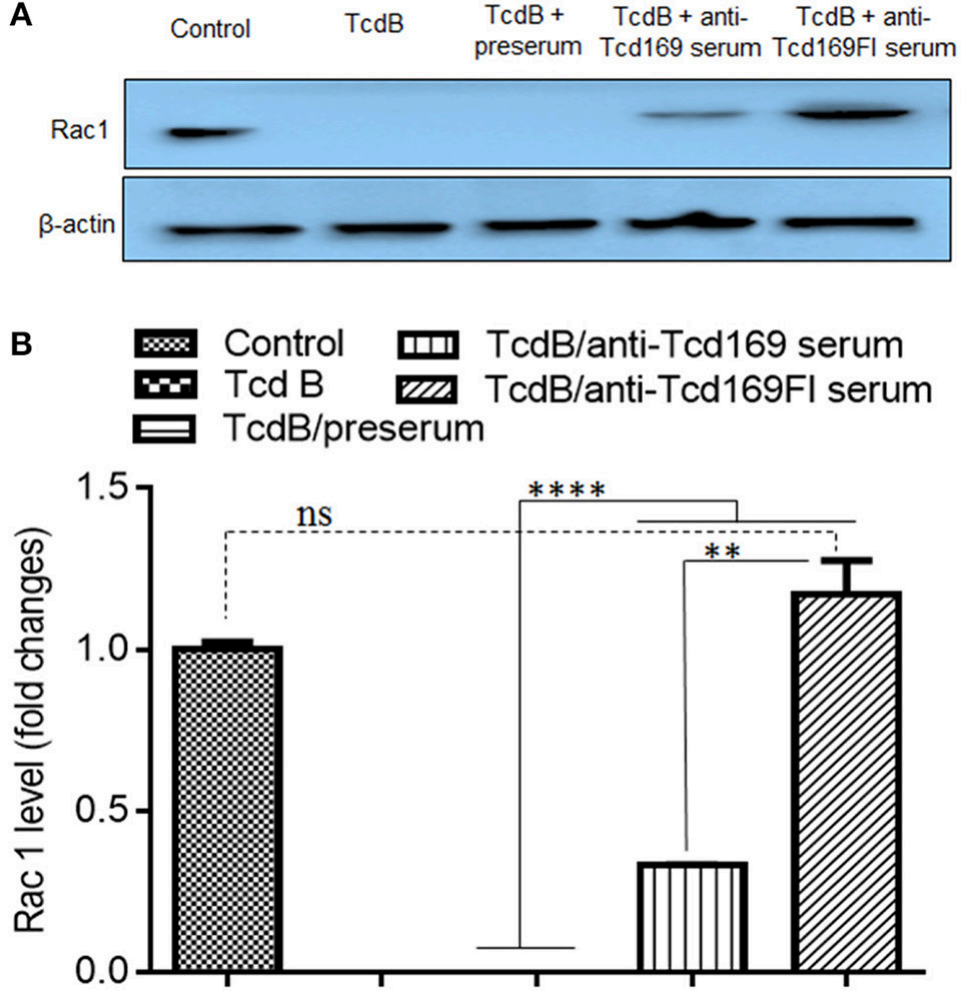
Glucosyltransferase activity of TcdB is blocked by anti-Tcd169 or anti-Tcd169FI serum. **(A)** CT26 cells were lysed, and the cytosolic fraction was exposed to TcdB (10 ng/ml) with or without serum for 1 h followed by Western Blot analysis using a monoclonal antibody that only recognizes non-glucosylated Rac1. β-actin was used as an equal loading control. **(B)** Quantitation of Rac1 levels in **(A)** (^**^p < 0.01, ^****^p < 0.0001, ns: no significant).

Since both Tcd169 and Tcd169Fl contain GTD and CPD domains of TcdB, we assessed whether anti-Tcd169 and anti-Tcd169Fl sera can inhibit GT and cysteine activities of TcdB. To this end, we first determined whether anti-Tcd169 and anti-Tcd169Fl sera can inhibit TcdB-mediated glucosylation/inactivation of Rac1 in CT26 cell lysates by Western blot analysis using an antibody only recognizing non-glucosylated Rac1. As shown in Figure 7, both anti-Tcd169 or anti-Tcd169Fl sera at 1:200 dilution significantly inhibited TcdB (10 ng/ml)-mediated glucosylation/inactivation of Rac1 in CT26 cell lysates. Interestingly, anti-Tcd169Fl serum was significantly more effective than anti-Tcd169 serum in blocking GT activity of TcdB (Figure 7), indicating that sFlic portion of Tcd169Fl can significant enhance anti-GTD responses.

**Figure 8:**
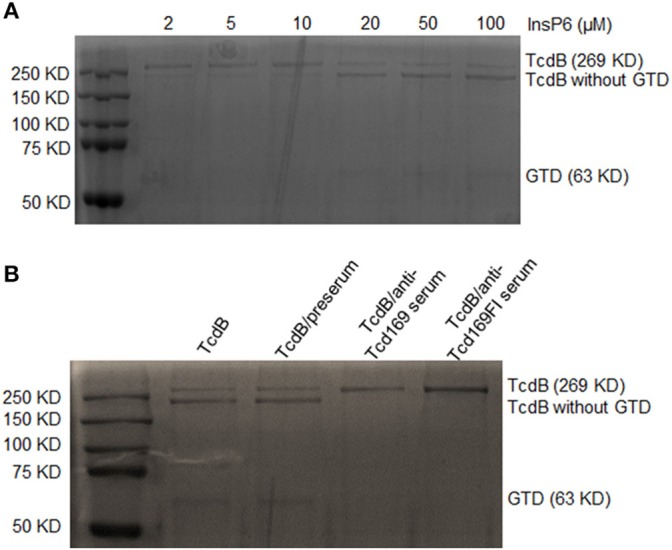
*In vitro* autocleavage of TcdB mediated by cysteine proteinase domain (CPD) is blocked by anti-Tcd169 or anti-Tcd169Fl serum. **(A)** SDS-PAGE of TcdB autocleavage in the presence of InsP6 (2–100 μM). **(B)** The activation of CPD-mediated TcdB autocleavage by InsP6 (20 μM) was blocked by anti-Tcd169 or anti-Tcd169FI serum. Full-length TcdB (269 KD) and TcdB without GTD (544–2366 aa), glucosyltransferase domain (GTD, 1–543 aa, 63KD) are indicated.

We then determined whether anti-Tcd169 and anti-Tcd169Fl sera can inhibit CPD-mediated autocleavage of TcdB. TcdB of 0.2 μg were incubated with InsP6, ranging in concentrations from 2 to 100 μM at 37°C for 1 h, and the reactions were stopped and resolved by SDS-PAGE. As shown in Figure 8A, InsP6 induced a dose-dependent autocleavage of TcdB. To examine if the anti-Tcd169 and Tcd169FI sera can inhibit the TcdB autoprocessing, sera at 1:200 dilution were added to the reaction system containing 20 μM of InsP6. As shown in Figure 8B, the TcdB autoprocessing was completely blocked by anti-Tcd169 or anti-Tcd169FI serum.

### Anti-Tcd169 or anti-Tcd169FI serum inhibits the binding of TcdA/TcdB to CT26 cells

**Figure 9:**
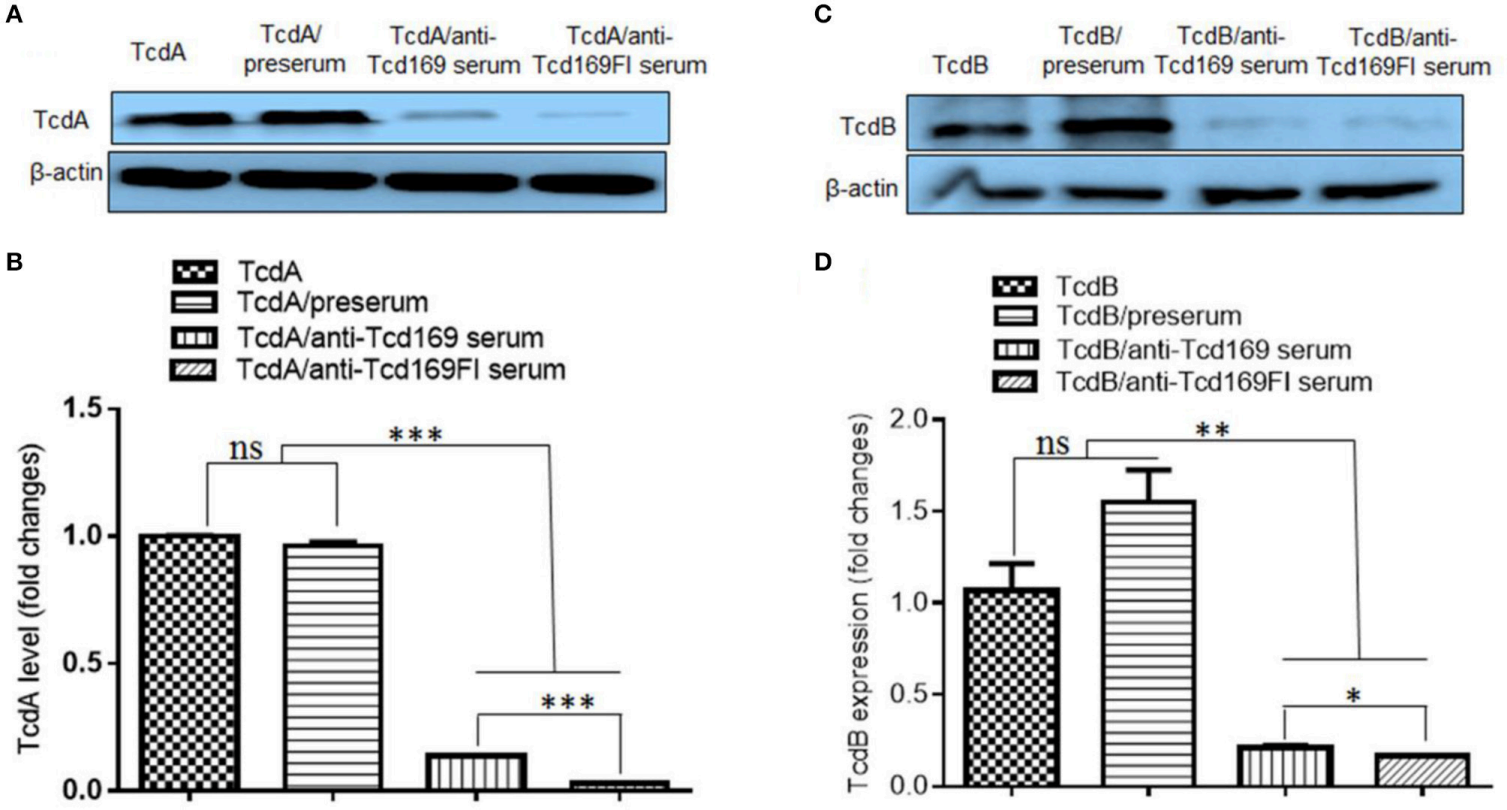
Anti-Tcd169 or anti-Tcd169Fl serum inhibits binding of TcdA/TcdB to CT26 cells. CT26 cells were exposed to TcdA **(A,B)** or TcdB **(C,D)** at 10 μg/ml with or without serum for 30 min on ice. Unbound toxins were removed by washing with PBS for three times. Cells bound with toxins were lysed and used for Western Blot analysis using anti-TcdA or anti-TcdB antibody. Preserum was used as control. Preserum, sera of Tcd169- or Tcd169FI-immumized mice were diluted at 1:200. **(B)** Quantitation of TcdA levels shown in **(A)**. **(D)** Quantitation of TcdB levels shown in **(D)** (^*^p < 0.05, ^**^p < 0.01, ^***^p < 0.001, ns: no significant).

Since both Tcd169 and Tcd169Fl contain RBD domains of TcdB and TcdA, we examined whether the binding of TcdA or TcdB to the CT26 cells is affected by the anti-Tcd169 or Tcd169Fl serum. CT26 cells were exposed to the TcdA or TcdB at 10 μg/ml with or without preserum or anti-Tcd169 or anti-Tcd169Fl serum at 4°C, for 30 min, after being washed three times, cells were collected for Western Blot analysis using anti-TcdA or anti-TcdB antibodies. As shown in Figure 9, both anti-Tcd169 and anti-Tcd169Fl sera at a dilution of 1:200 significantly inhibited bindings of both TcdA and TcdB to CT26 cells, while the anti-Tcd169Fl serum was significantly more effective in blocking the bindings of TcdA and TcdB to CT26 cells, suggesting that sFlic portion of Tcd169Fl can significant enhance anti-RBD responses.

### Tcd169/Tcd169Fl vaccinations protect mice against infection with an epidemic *C. difficile* strain

**Figure 10:**
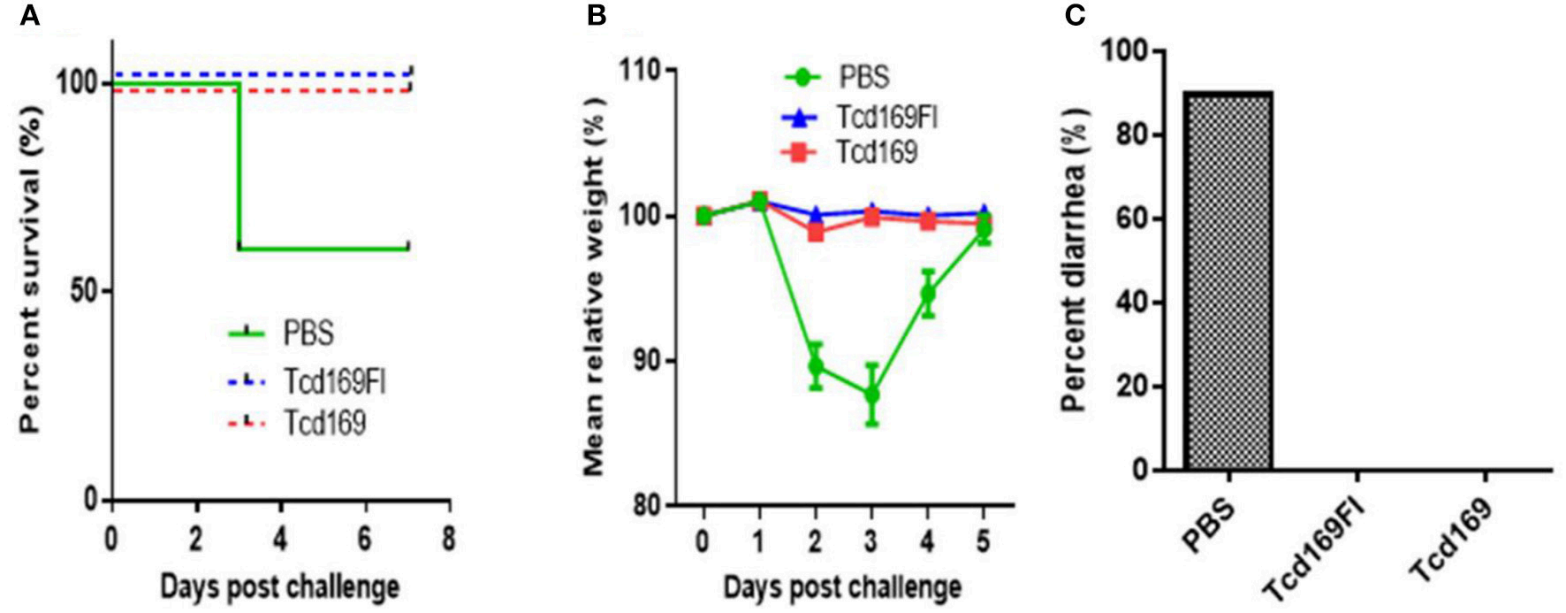
Tcd169 or Tcd169FI immunization via i.m. route provides mice full protection against infection with *C. difficile* strain UK1. Mice (*n* = 10) were challenged with *C. difficile* UK1 spores (10^6^/mouse) 14 days after the third immunization with Tcd169, Tcd169Fl, or PBS. Kaplan-Meier survival plots (*P* = 0.002754 between PBS-immunized and the 2 immunized groups) **(A)**, mean relative weight of all surviving mice (up to the day of death) **(B)**, and frequency of diarrhea **(C)** of different groups were illustrated. Data are presented as mean ± SD (*n* = 10).

We further evaluated the protection efficacy of Tcd169, Tcd169Fl in a mouse model of CDI. After three immunizations via i.m., mice were challenged with 10^6^ spores of *C. difficile* UK1. Approximately 40% PBS-immunized mice died or became moribund and were euthanized by day 3 post-infection (Figure 10A). In contrast, Tcd169, Tcd169FI-immunized mice showed no appreciable signs of disease (Figure 10). Nine of ten mice in PBS-immunized mice developed weight loss (Figure 10B) and diarrhea (Figure 10C).

### Immunizations of mice with Tcd169 or Tcd169Fl decrease *C. difficile* spores and toxin levels in the feces after infection

**Figure 11:**
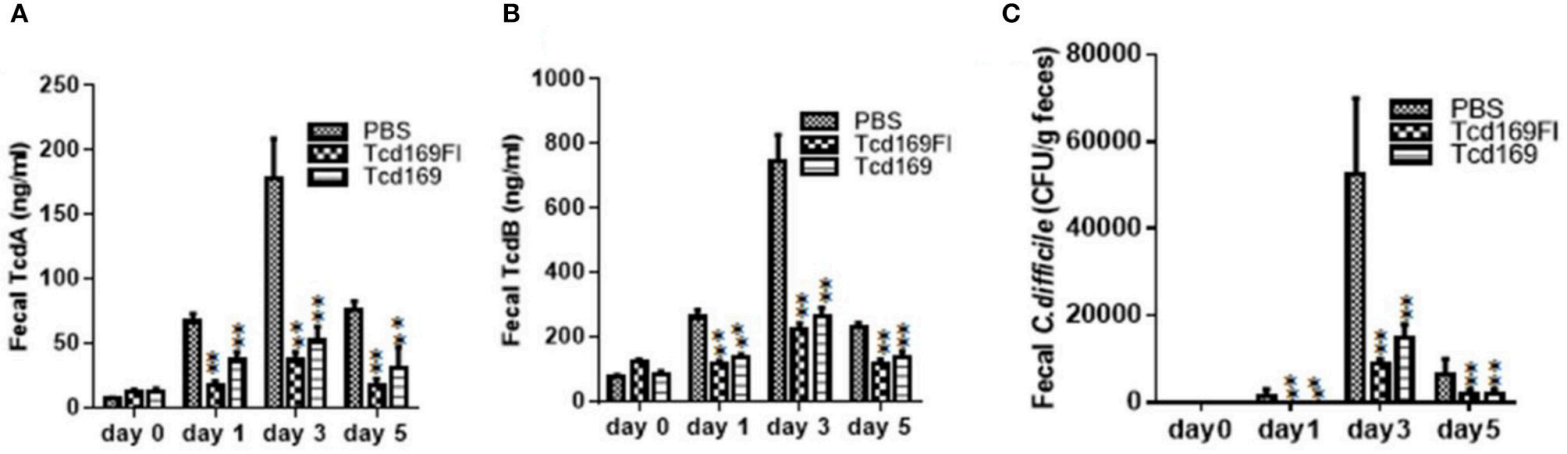
Immunizations of mice with Tcd169 or Tcd169Fl decrease numbers of *C. difficile* spores and toxin levels in the feces after infection with *C. difficile* spores. *C. difficile*-challenged mice were monitored for fecal TcdA **(A)** and TcdB **(B)** levels or *C. difficile* spore shedding **(C)**. Data are given as mean ± SD (^**^P < 0.01 vs. PBS; *P* < 0.05 in **(A)** day 1 and in **(C)** day 3 between Tcd169 and Tcd 169FI groups, others have no difference between Tcd169 and Tcd 169FI groups, *P* > 0.1).

Immunization of mice with Tcd169 and Tcd169Fl significantly decreased TcdA (Figure 11A) and TcdB (Figure 11B) concentrations, and the spore count (Figure 11C) in feces, in comparison with PBS-immunized group. In addition, immunization of mice with Tcd169Fl significantly decreased spore count in feces, in comparison with Tcd169-immunized mice (Figure 11C), indicating that sFliC portion of the Tcd169Fl may stimulate immune responses targeting *C. difficile* colonization. In fact, Tcd169Fl was able to induce much stronger anti-sFlic IgA responses than anti-TcdA/anti-TcdB IgA responses (Figure 4).

### Tcd169Fl stimulates TLR5 activation

**Figure 12:**
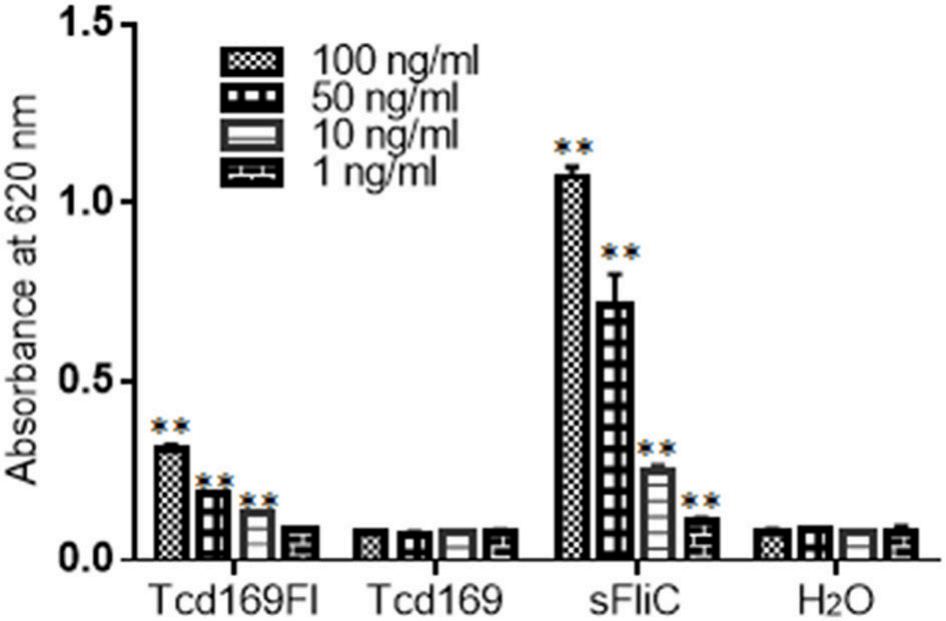
Tcd169Fl activates mTLR5. Activation of mTLR5 by sFLic/Tcd169Fl was evaluated in an mTLR5 reporter assay at concentrations of 100, 50, 10, and 1 ng/ ml. Each sample was analyzed in triplicate, and data were given as mean ± SD (^**^P < 0.01 vs. H_2_O group).

Toll-like receptor 5 (TLR5) is known to recognize bacterial flagellin from invading mobile bacteria (47). By using a murine model, Jarchum et al. (48) showed that TLR5 stimulation protects mice from acute *C. difficile* colitis. To investigate whether Tcd169Fl can activate TLR5, we performed the TLR5 reporter assay. As shown in Figure 12, Tcd169Fl still activated TLR5 at concentrations of 100, 50, and 10 ng/ml, while Tcd169 did not.

## Discussion

We generated a new chimeric protein, Tcd169, by fusing GT, CPD, and RBD of TcdB and RBD of TcdA. We further fused Tcd169 with sFliC, generating Tcd169FI. Immunization of mice with Tcd169 or Tcd169Fl induced protective immunity against TcdA/TcdB challenge through intraperitoneal injection, also provided mice full protection against infection with a hyper-virulent *C. difficile* strain (BI/NAP1/027). Our results showed that immunizations with Tcd169 or Tcd169Fl could: (1) induce both Th1 and Th2 responses while at different extent (Figure 5); and (2) induce protective immune responses against all toxin domains included in the two fusion proteins (Figures 7–9). Interestingly, our data suggest that inclusion of sFlic in the fusion protein (Tcd169Fl) can significantly enhance its protective immunity, when compared with Tcd169, by (1) inducing significantly more anti-TcdB IgGs and anti-TcdA IgG2b (Figure 5A); (2) inducing significantly more anti-GTD of TcdB (Figure 7) and anti-RBD of TcdB/TcdA (Figure 9) antibodies; (3) inducing more anti-TcdA neutralizing antibodies *in vivo* (Figure 6); (4) reducing *C. difficile* dissemination and TcdA/TcdB levels in feces from Tcd169Fl-immunized mice infected *C. difficile* in comparison with Tcd169-immunized mice (Figure 11); and (5) stimulating TLR5 activation, though at a reduced level in comparison with sFlic stimulation alone (Figure 12).

Overall, our results are in agreement with previous reports by other groups showing that sFliC is able to enhance the immunogenicity of immunogens (30, 49) and that administration of purified *Salmonella*-derived flagellin, a Toll-like receptor 5 (TLR5) agonist, protects mice from *C. difficile* colitis by delaying *C. difficile* growth and toxin production (48). There are a few slight data discrepancies, i.e., we did not find significant differences in anti-TcdA/TcdA IgG titers (Figure 3) and *in vitro* anti-TcdA/TcdB neutralizing titers (Figure 4) between anti-Tcd169 and anti-Tcd169Fl sera as observed in other experiments, which might be due to the sensitivities of the methods used. In addition, we observed sFliC-mediated enhanced *in vivo* anti-TcdA, but not anti-TcdB, neutralizing activity (Figure 6). The sFliC-mediated enhancement of anti-TcdB neutralizing activity might be covered by the TcdB dosage effect.

We included C-termial regions of TcdA (aa 1848–2710) and TcdB (aa 1851–2366) in two fusion proteins Tcd169 and Tcd169Fl. Historically, these regions are called combined repetitive oligopeptides (CROP), and were considered RBD for TcdA and TcdB (50, 51). Recently, two binding sites were postulated within the newly defined RBD of TcdB. TcdB region aa 1372–1493 is bound by PVRL3 and TcdB region aa 1501–1830 by FZD receptor proteins, respectively, whereas TcdB CROP region (1851–2366) is bound by CSPG4 (52, 53). The recpetor binding regions of TcdA and corresponding receptors are less clear so far. It was reported that TcdA could interact with different surface carbohydrate structures and with two proteins (sucrase-isomaltase and glycoprotein gp96) (54). Our data showed that anti-Tcd169 or anti-Tcd169 serum could dramatically reduced and dimished bindings of both TcdA and TcdB to CT26 cells, indicating that CROP regions of TcdA/TcdB are still the major receptor binding sites with other reported/postulated binding sites as adjunctive niches for additional recepor bindings.

In the future, we will evaluate the effects of immunization dosages on antibody responses, and further evaluate the protecive efficay of Tcd169 and Tcd169Fl in hamster model of CDI.

## Author contributions

XS designed the project and participated in data analysis. SW, YW, and YC performed experiments. SW, YW, XS, and CK participated in data analysis and wrote the manuscripts. All authors read and approved the final manuscript.

### Conflict of interest statement

The authors declare that the research was conducted in the absence of any commercial or financial relationships that could be construed as a potential conflict of interest.
